# Advanced prostate cancer diagnosed by bone metastasis biopsy immediately after initial negative prostate biopsy: a case report and literature review

**DOI:** 10.3389/fonc.2024.1365969

**Published:** 2024-05-10

**Authors:** Mingwen Liu, Zhifei Xie, Wen Tang, Guobiao Liang, Zeju Zhao, Tao Wu

**Affiliations:** Department of Urology, The Affiliated Hospital of Zunyi Medical University, Zunyi, Guizhou, China

**Keywords:** prostate cancer, metastatic prostate cancer, prostate biopsy, metastatic biopsy, repeated puncture of prostate

## Abstract

Prostate cancer (PCa) is a prevalent male malignancy that originates in the epithelial cells of the prostate. In terms of incidence and mortality of malignant tumors in men, PCa ranks second and fifth globally and first and third among men in Europe and the United States, respectively. These figures have gradually increased in recent years. The primary modalities used to diagnose PCa include prostate-specific antigen (PSA), multiparametric magnetic resonance imaging (mpMRI), and prostate puncture biopsy. Among these techniques, prostate puncture biopsy is considered the gold standard for the diagnosis of PCa; however, this method carries the potential for missed diagnoses. The preoperative evaluation of the patient in this study suggested advanced PCa. However, the initial prostate puncture biopsy was inconsistent with the preoperative diagnosis, and instead of waiting for a repeat puncture of the prostate primary, we performed a biopsy of the rib metastasis, which was later diagnosed as advanced PCa.

## Background

PCa is a highly common malignant tumor in males that originates from the prostate’s epithelial cells. Prostate puncture biopsy is thought to be the gold standard for identifying PCa. However, this method carries the potential for missed diagnoses. Usually, when this occurs, a repeat puncture of the prostate is required. This article describes a patient with advanced prostate cancer who underwent an initial prostate puncture biopsy with negative results. Instead of performing a repeat biopsy of the prostate, we performed a puncture of the rib metastases and succeeded in achieving the expected results.

## Case presentation

**Figure 1:**
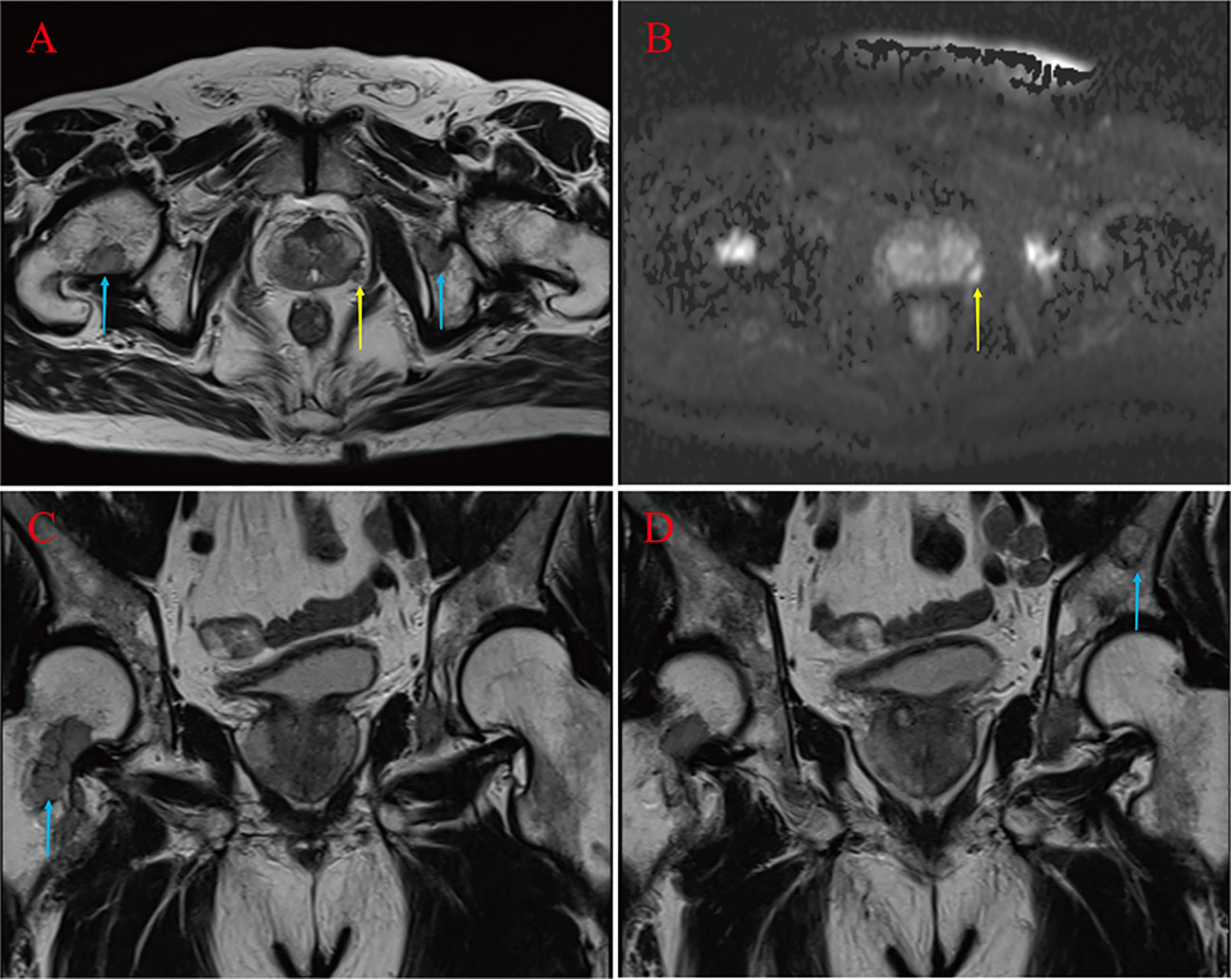
Suspicious primary focus at 4 points in the left lobe of the prostate gland (yellow arrow) **(A, B)**. Right femoral neck, left pubic bone and sciatic metastases (blue arrow) **(A, C, D)**.

**Figure 2:**
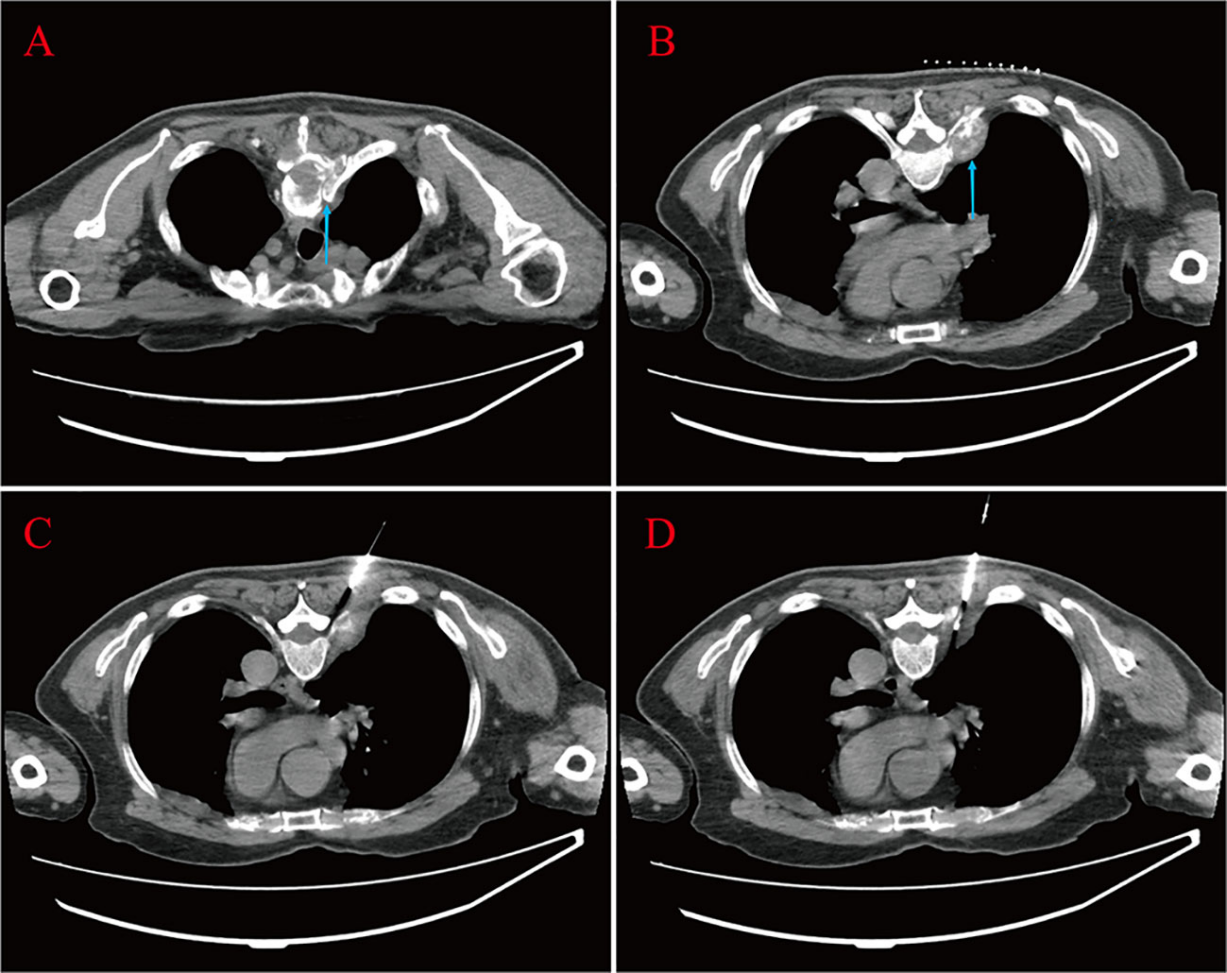
Bone destruction of the thoracic vertebrae and soft tissue mass formation at the root of the ribs are seen (blue arrow) **(A, B)**. The patient lay prone on the CT examination bed; one strip of puncture tissue was taken and sent for analysis **(C, D)**.

A 67-year-old male was admitted to the hospital in April 2023. The patient had repeated low back pain for two years (VRS pain assessment grade III), was unable to stand and relied on a wheelchair to get around, and oral painkillers were ineffective; there were no apparent symptoms of urinary tract irritation or hematuria, and nocturia was present 2-3 times/night. Rectal examination digitalized (DRE): Prostate II° large, hard, no suspicious nodules were palpated. The patient was diagnosed with essential hypertension grade 2 (very high-risk group) and type 2 diabetes mellitus 20 years ago and underwent coronary balloon dilatation for coronary artery disease two weeks before admission. The patient had no history of smoking or alcohol abuse, no history of urological disease, and no family history of hereditary disease. After admission, relevant laboratory tests showed serum total prostate-specific antigen (TPSA) 528.671 ng/ml, free prostate-specific antigen (FPSA) 38.770 ng/ml, and the ratio of free PSA to total PSA (%fPSA) 0.073. PSA density (PSAD): 13.1353 ng/ml^2^ (the volume of the prostate’s calculation: height×width×length×0.52). Imaging results: prostate MRI scanning + enhancement: prostate central band and migratory band were enlarged, prostate signal was uneven, prostate size was about 3.6 cm×5.0 cm×4.3 cm. T2WI showed: prostate left lobe at 4 o’clock, see a diameter of about 0.6cm small round slightly shorter T2 signal (**Figures 1A–D**). Prostate Imaging (Prostate Imaging and Data System (PI-RADS) score: 4 points for T2WI, 5 points for DWI, 5 points for DCE, and 5 points for the overall score, which was a PCa nodule (T2N2M1b). Thoracic CT: multiple bone destruction and some soft tissue mass formation in the thoracic spine area, and metastatic tumor lesions were considered (metastatic foci depicted in **Figures 2A, B**).

**Figure 3:**
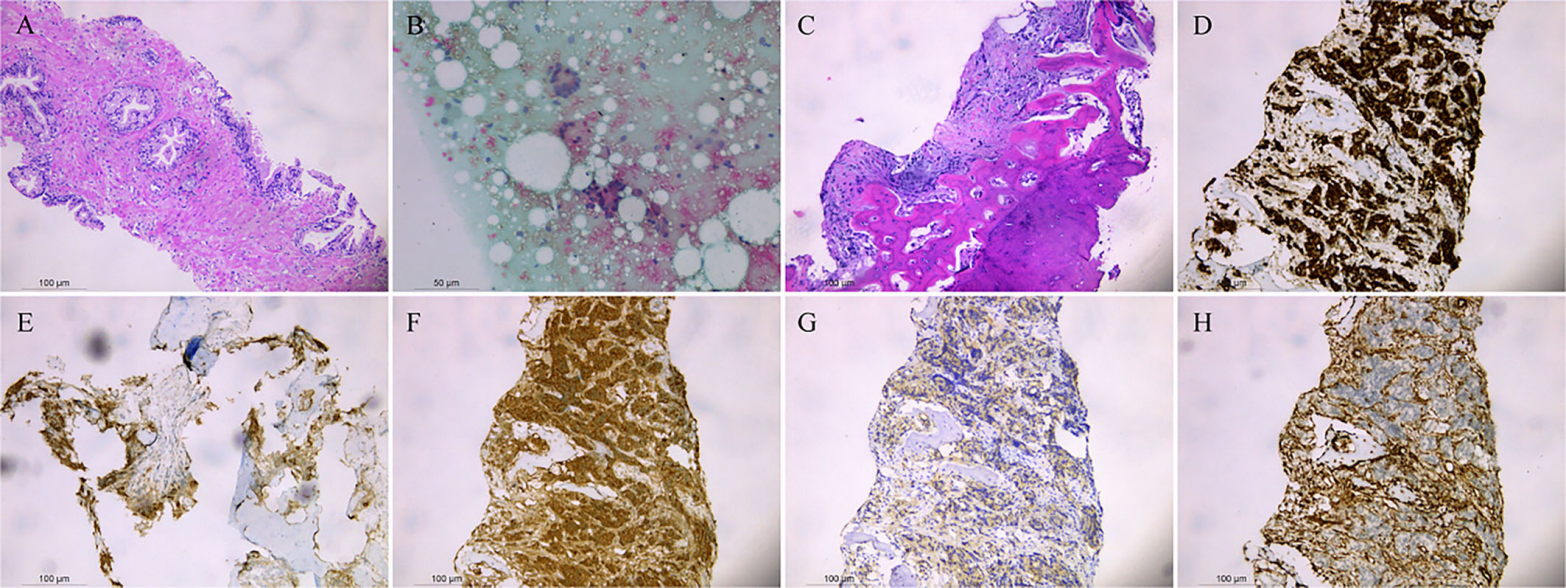
Benign prostatic hyperplasia was observed in the left and right lobes **(A)**. Punctured tissue smear shows daisy-like heterogeneous cell clusters **(B)**. Prostate follicular carcinoma, CK (+), P504S (+), PSA (+), PSAP (+), and Vimentin (-) are shown by immunohistochemistry **(C–H)**.

The above findings were highly suggestive of advanced PCa with multiple bone metastases, so with the consent of the patient and his family, we performed a transperineal prostate biopsy (TPBx) based on the anatomical structure of the prostate and the suspicious foci as shown in the MRI. Nine cores were taken from the suspected primary lesion on the left side, and prostate tissue was taken from the right side using the same method. Postoperative pathological findings showed (left and right lobes) benign prostatic hyperplasia (BPH); immunohistochemical staining showed epithelial cells: CK-H, p63 (indicating the presence of myoepithelium), and P504S (luminal margin +) (**Figure 3A**). The pathological findings were inconsistent with the clinical diagnosis, because the initial visual alignment targeted biopsy had been used, and the number of biopsy cores (18 stitches) was close to saturation, after thoroughly evaluating the patient’s physical condition and obtaining the patient’s consent, we did not perform a repeat biopsy of the prostate gland but instead performed a CT-guided biopsy of the rib metastases in the chest of the patient. The patient was lying prone on the CT examination bed, and the CT was localized to the metastasis at the root of the right 7th rib (**Figures 2C, D**), and one strip of puncture tissue was obtained and sent for examination. Postoperative immunohistochemical results: prostate follicular carcinoma, Gleason score: 5 + 4, CK (+), P504S (+), PSA (+), PSAP (+), and wave protein (-), suggestive of metastatic PCa (**Figures 3B–H**).

The patient was diagnosed with advanced metastatic prostate cancer (cT2N2M1b) with no indication for surgery. To alleviate the patient’s clinical symptoms, slow down the tumor progression, and improve the prognosis, he was given Rezvilutamide Tablets 240mg orally, denosumab 120 mg, and Goserelin 3.6mg. Review after one month: TPSA 14.293 ng/ml, PSA decreased to normal in 3 months, and now it has been followed up for ten months, TPSA 0.023 ng/ml, testosterone (TES) <0.087 nmol/L. The patient actively cooperated with the treatment during the medication period, and there were no adverse and unanticipated events. The patient’s bone pain symptoms improved significantly (VRS pain assessment grade I), and he could walk independently without urination abnormalities. The patient is now on endocrine therapy and undergoes regular follow-ups with a good life status.

## Discussion and conclusions

Originating from the prostate’s epithelial cells, PCa is one of males’ most prevalent malignant tumors. The GLOBOCAN 2020 data indicate that the incidence and mortality rates of PCa ranked second and fifth among malignant tumors among men globally and first and third among men in Europe and the US, respectively. PCa has been increasingly common in recent years (1).The incidence of advanced PCa is higher in all Asian countries except Japan, which is directly related to the screening of high-risk populations in Japan. The prognosis of PCa also depends on the quality of treatment; low reimbursement rates for some medications used to treat advanced PCa and patient delays caused by financial hardship will further lower the survival rate. Regular screening aids in the early diagnosis of PCa and improves the 5-year survival rate (2).

The PSA is a crucial marker for PCa screening; the higher the level, the greater the probability of PCa present (3, 4). However, since false positives can result from various conditions (prostatitis, catheterization, etc.), the combination of PSAD and %fPSA is a more reliable indicator of PCa presence. While the chance of prostate inflammation diminishes with increasing PSA density, the actual probability of having PCa increases; 56% of men with %fPSA < 0.10 had PCa (5, 6). With the rapid development of imaging technology, MRI plays an increasingly important role in the diagnosis of PCa. Performing an MRI before a biopsy can increase the cancer detection rate and reduce the number of unnecessary biopsies to a certain extent, even in patients with previous negative biopsies (7, 8). Puncture biopsy of the prostate is the gold standard for the diagnosis of PCa, both by transrectal and transperineal routes; however, the sensitivity are not significant, with the detection rate of PCa ranging from 28-56.9 percent with a standard 12-core systematic biopsy (9–11). Moreover, it has been documented that up to 30% of serious prostate tumors are missed by a typical biopsy strategy consisting of 10–12 cores (12). In contrast, a transrectal saturated prostate biopsy (greater than 20 cores) is more likely to identify severe PCa and prevent the need for additional biopsies (13). The incidence of infection-related complications and rectal bleeding is relatively low due to the transperineal route, which avoids the rectal mucosa, and the cancer detection rate does not differ from that of transrectal puncture (14). However, additional subgroup analysis has demonstrated that when PCa staging is in the T1–T2 stage, the biopsy detection rate of TPBx is greater (15). If the patient is a first-time recipient of a transperineal route puncture, the PCa detection rate is significantly higher when 18 cores are taken than when 6-12 cores are taken in a protocol (16). Performing MRI-targeted biopsies can improve cancer detection, and there are currently three strategies: in-bore targeted biopsies, visual registration, and software-based MRI/ultrasound image-fusion systems. There was no significant difference in cancer detection rates between the three modalities, with visual registration being the most cost-effective targeted biopsy method (17). PCa detection rates between cognitive registration and MRI/ultrasound image-fusion targeting techniques have been further analyzed in one report, with no significant difference between the methods of transperineal route puncture (18). Inevitably, however, there is a possibility of a missed diagnosis even when combined with an MRI. In this case, the patient had undergone an MRI, and the surgeon performed a cognitively aligned targeted biopsy. The pathological result was BPH, which was inconsistent with the patient’s clinical presentation and serological and imaging findings. The patient’s serum PSA was significantly elevated, and the imaging was highly suspicious of advanced PCa with indications for repeat biopsy (19). Furthermore, the patient had an elevated PSAD, which is crucial for determining if PCa will be present in individuals who have had negative biopsies in the past (5). Therefore, further examination to clarify the diagnosis was necessary.

The best timing to conduct a second biopsy is still up for debate, however reports indicate that the higher the detection rate, the later the repeat biopsy should be performed (19). Repeat biopsies can also be combined with MRI for a targeted prostate biopsy to improve cancer detection and help doctors confirm the diagnosis, but generally, they need to wait for local inflammation and edema in the prostate to subside according to the principles of treatment (20–22). Furthermore, patients may have increased anxiety and a higher likelihood of complications from repeat prostate biopsies, which could result in lower compliance (23, 24). Due to the high incidence of advanced PCa in China, if the pathological results of repeated puncture are still inconsistent with the clinical diagnosis, it may not be possible to accurately formulate a diagnostic and therapeutic plan, which may affect the patient’s prognosis. Bone is the most common metastatic site of advanced PCa (25); therefore, after fully communicating with the patient and obtaining the patient’s consent, we performed a CT-guided biopsy of the rib metastasis, and the postoperative pathological results were consistent with the clinical diagnosis. After treatment, the patient’s bone pain symptoms have been significantly improved, and the PSA at three months has been reduced to normal. In addition, the patient’s treatment cost has been reimbursed by most of the patient’s expenses, which has alleviated the patient’s financial and psychological burden and improved the patient’s quality of life.

In our opinion, when the patient’s various examination results have been highly suggestive of advanced PCa with distant metastases and the initial prostate puncture biopsy has been combined with MRI but the pathological results are still negative; there is no need to wait for a repeat biopsy of the primary focus, and a CT-guided puncture biopsy of metastatic foci can be carried out if the patient’s physical status allows it. This technique can be performed under CT guidance without the need for compatible equipment related to MRI technology, saving costs, and no postoperative complications such as infections, bleeding, and other related complications have been observed. However, this technique is relatively difficult and requires a high level of operator proficiency. In conclusion, biopsy of metastases can be a new option in advanced prostate patients in whom MRI-targeted biopsy has been implemented, if the diagnosis is still not confirmed.

## Data availability statement

The original contributions presented in the study are included in the article/supplementary material. Further inquiries can be directed to the corresponding authors.

## Ethics statement

The study was reviewed and approved by the Ethics Committee of the Affiliated Hospital of Zunyi Medical University. Written informed consent was obtained from the individual(s) for the publication of any identifiable images or data included in this article.

## Author contributions

ML: Writing – original draft. ZX: Writing – original draft. WT: Writing – review & editing. GL: Writing – review & editing. ZZ: Writing – review & editing. TW: Writing – review & editing.
